# A Novel Spiro-Heterocyclic Compound Identified by the Silkworm Infection Model Inhibits Transcription in *Staphylococcus aureus*

**DOI:** 10.3389/fmicb.2017.00712

**Published:** 2017-04-25

**Authors:** Atmika Paudel, Hiroshi Hamamoto, Suresh Panthee, Keiichi Kaneko, Shigeki Matsunaga, Motomu Kanai, Yutaka Suzuki, Kazuhisa Sekimizu

**Affiliations:** ^1^Institute of Medical Mycology, Teikyo UniversityHachioji, Tokyo, Japan; ^2^Laboratory of Synthetic Organic Chemistry, Graduate School of Pharmaceutical Sciences, The University of TokyoTokyo, Japan; ^3^Department of Medical Genome Science, Graduate School of Frontier Sciences, The University of TokyoKashiwa, Japan; ^4^Genome Pharmaceuticals Institute Co., Ltd.Bunkyo, Tokyo, Japan

**Keywords:** chemical library, antimicrobial agent, therapeutic activity, anti-staphylococcal, silkworm infection model, RNA polymerase, sigma factor

## Abstract

Synthetic compounds are a vital source of antimicrobial agents. To uncover therapeutically effective antimicrobial agents from a chemical library, we screened over 100,000 synthetic compounds for *in vitro* antimicrobial activity against methicillin-resistant *Staphylococcus aureus* and evaluated the *in vivo* therapeutic effectiveness of the hits in *S. aureus*-infected silkworms. Three antimicrobial agents exhibited therapeutic effects in the silkworm infection model. One of these, GPI0363, a novel spiro-heterocyclic compound, was bacteriostatic and inhibited RNA synthesis in *S. aureus* cells. GPI0363-resistant *S. aureus* strains harbored a point mutation in the gene encoding the primary sigma factor, SigA, of RNA polymerase, and this mutation was responsible for the resistance to GPI0363. We further revealed that GPI0363 could bind to SigA, inhibit promoter-specific transcription *in vitro*, and prolong the survival of mice infected with methicillin-resistant *S. aureus*. Thus, GPI0363 is an attractive candidate therapeutic agent against drug-resistant *S. aureus* infections.

## Introduction

Infectious diseases are a major cause of disease and death around the world. The emergence of drug-resistant pathogens has drastically increased, resulting in a scarcity of effective antimicrobials. The worldwide spread of multidrug-resistant pathogens has greatly increased the economic and health burden (WHO, 2014). New antimicrobials with therapeutic effectiveness against drug-resistant pathogens are thus urgently needed. *Staphylococcus aureus* is a Gram-positive opportunistic pathogen that causes infectious diseases such as sepsis and pneumonia, and skin and soft tissue infections (Lowy, 1998). *S. aureus* strains resistant to clinically used antibiotics such as methicillin, linezolid, daptomycin, and vancomycin are a serious threat to global health (Jevons, 1961; Hiramatsu et al., 1997; Tsiodras et al., 2001; Mangili et al., 2005; Marty et al., 2006; Howden et al., 2010). The discovery and development of novel antibacterial agents effective against such drug-resistant staphylococci is therefore critical.

The screening of synthetic compounds for antimicrobial activities began with the discovery of arsphenamine by Paul Ehrlich in 1907 (Williams, 2009). The introduction of sulfanilamides, quinolones, and linezolid, among others, demonstrated that chemically synthesized molecules are promising for the treatment of infectious diseases caused by pathogenic bacteria (Emmerson and Jones, 2003; Shaw and Barbachyn, 2011; Aminov, in press). Of the 30 new antimicrobials launched since 2000, more than 50% are synthetic compounds, including two new classes of antimicrobial agents (Butler et al., 2017). Additionally, 14 synthetic antimicrobials are currently in phase II or phase III clinical trials (Butler et al., 2017). Therefore, synthetic compounds comprise a major and promising source of antimicrobial agents, and the screening of chemical libraries can reveal promising drug leads for the treatment of infectious diseases.

Apart from finding new compounds effective against drug-resistant pathogens, obtaining therapeutically effective agents for clinical application is crucial. This is a challenging task as not all agents with *in vitro* effectiveness are effective *in vivo* due to factors such as poor pharmacokinetic parameters and toxicity, which are difficult to predict based simply on the chemical structure. Individual assessment of the therapeutic activity of each newly discovered compound is therefore essential. The identification of therapeutically effective agents requires screening in animal models. The use of mammalian models for screening, however, is fraught with ethical issues in addition to high cost and the need for sophisticated facilities. The use of invertebrate models, on the other hand, avoids such issues, and is highly suitable for the early stages of screening and drug development.

In the present study, we utilized a silkworm infection model to screen a chemical library of synthetic compounds for the first time, and identified a spiro-heterocyclic molecule, GPI0363, as a novel anti-staphylococcal agent. We demonstrated that GPI0363 inhibited RNA synthesis *in vivo* in *S. aureus*, and found that the primary sigma factor, SigA, was involved in its anti-staphylococcal activity.

## Materials and methods

### Chemical library screening

#### Primary screening

The chemical library of the Drug Discovery Initiative at the University of Tokyo (http://www.ddi.u-tokyo.ac.jp/en/) was used. As of March 2016, this library comprises more than 230,000 “synthetic compounds regulating a biological function.” Chemical compounds obtained from the library were dissolved in dimethyl sulfoxide (DMSO) at a final concentration of 10 mM. Inhibition of MRSA growth was determined by broth microdilution assay (Clinical and Laboratory Standards Institute, 2012). Briefly, *S. aureus* MRSA4 was grown with aeration in 5 mL Tryptic Soy Broth (TSB; Becton Dickinson and Company, Franklin Lakes NJ, USA) in a shaker maintained at 37°C. The overnight culture was diluted with cation-adjusted Muller–Hinton Broth (MHB; Becton Dickinson and Company) to have ~5 × 10^5^ colony forming units (CFU)/mL per well in a round bottom 96-well-plate. Compounds were added to each well to obtain a final concentration of 100 μM. Vancomycin was used as a positive control and DMSO as a vehicle control in each plate. The plates were incubated at 37°C for 20 h. Compounds that inhibited growth when vancomycin inhibited the growth and DMSO did not inhibit the growth were selected as hits.

#### Secondary screening

Therapeutic activity in silkworms infected with methicillin-susceptible *S. aureus* (MSSA) was determined as previously described (Hamamoto et al., 2015). Briefly, hatched larvae from silkworm eggs (Hu•Yo × Tsukuba•Ne, Ehime Sanshu, Japan) were fed and grown at 27°C until the fourth molt stage. *S. aureus* MSSA1 was grown with aeration in 5 mL TSB at 37°C. The overnight culture was diluted with 0.9% NaCl to obtain ~6 × 10^8^ CFU/mL. Fifty microliters of the culture was injected into the hemolymph of fifth instar 2nd-day larvae (*n* = 3). Compounds (10 mM) dissolved in DMSO were diluted with 0.9% NaCl to obtain a concentration of 2 mM, thus producing a final DMSO concentration of 20%. Silkworms were immediately injected with 50 μL of each compound separately and larvae were further incubated at 27°C without feeding. Control groups received *S. aureus* or 20% DMSO in 0.9% NaCl or *S. aureus* and vancomycin (10 μg/larva). Silkworm survival was judged on day 2 when all the silkworms injected with only bacteria died, those injected with vehicle survived, and those injected with bacteria and vancomycin survived. Compounds that cured at least 2 of 3 silkworms at day 2 post-injection were judged to be therapeutically effective. To determine the half-maximal effective dose (ED_50_), compounds dissolved in DMSO were diluted with 0.9% NaCl; 50 μL of which was injected into the hemolymph of the silkworm (*n* = 10) after injection of *S. aureus*. The final concentrations of the compounds were 200, 150, 100, 75, 50, 25, and 12.5 μg/larva. Survival was plotted against the concentration, and the concentration that allowed for 50% silkworm survival was calculated from the graph.

### Antimicrobial spectrum

Bacterial cultures were prepared in either Luria Bertani medium (tryptone 10g/L, yeast extract 5g/L, NaCl 10g/L, pH 7.0), TSB, or MHB. Cation-adjusted MHB was used for antimicrobial susceptibility tests. For streptococcus species, cation-adjusted MHB with 2.5% lysed horse blood (Nippon Biotest Laboratories Inc, Tokyo, Japan) was used. The minimum inhibitory concentration (MIC) was determined by broth microdilution assay (Clinical and Laboratory Standards Institute, 2012).

### Bacteriostatic activity

The bacteriostatic activity was tested according to the National Committee for Clinical Laboratory Standards guidelines (National Committee for Clinical Laboratory Standards, 1999). Briefly, overnight culture of *S. aureus* MSSA1 grown at 37°C in MHB was diluted 1,000 times in MHB and cultured for 2 h at 37°C. For daptomycin, MHB was supplemented with 50 mg/L Ca^2+^. GPI0363 (20 μg/mL) or daptomycin (5 μg/mL) was added to 1 mL of the culture and incubated for 24 h at 37°C. Culture aliquots were collected at the indicated time, diluted, spread on Luria Bertani agar plates, and incubated for 24 h at 37°C. Cell viability was determined by counting the CFU of bacteria per milliliter. The lower limit of detection was 10^4^ CFU/mL. Data were analyzed using Prism 5 for Mac OS X, version 5.0d (GraphPad Software).

### Incorporation of radiolabeled *N*-acetyl-glucosamine, uridine, thymidine, and methionine

The amount of incorporated radiolabeled precursors was measured as previously described (Maki et al., 2001; Paudel et al., 2012, 2013). *S. aureus* RN4220 was grown to exponential phase and used for the assay in the presence of 25 μci [^3^H] *N*-acetyl-glucosamine (American Radiolabeled Chemicals, St. Louis, MO, USA) or 2 μci/mL of either [^3^H] uridine (Moravek Biochemical, Brea, CA, USA), [^3^H] thymidine (Moravek Biochemical), or [^35^S] methionine (Institute of Isotopes, Budapest, Hungary). Vancomycin, rifampicin, norfloxacin, and chloramphenicol (100 μg/mL each) were used as inhibitors of peptidoglycan, RNA, DNA, and protein synthesis, respectively. Vancomycin (100 μg/mL) or ampicillin (100 μg/mL) and vehicle were used as controls. GPI0363 (125 μg/mL) was used for all the assays. Aliquots were collected at 5, 10, 15, 20, and 30 min. Radioactivity of the acid insoluble fraction was counted with a liquid scintillation counter (LS6000SE, Beckman Coulter, Carlsbad, CA, USA) and expressed as counts per minute (CPM). Data were analyzed using Prism 5 for Mac OS X, version 5.0d (GraphPad Software).

### Isolation and analysis of *S. aureus* mutants resistant to GPI0363

*S. aureus* RN4220 was treated with 0.2% ethyl methanesulfonate overnight, spread on Luria Bertani agar plates containing 12.5, 25, and 50 μg/mL GPI0363, and incubated overnight at 30°C. The isolated strains were further grown at two temperatures, 30°C and 43°C. Strains that were viable at 30°C and non-viable at 43°C were defined as temperature-sensitive strains. Temperature-sensitive GPI0363-resistant strains were selected for whole-genome sequencing. The genome was sequenced according to previous report (Panthee et al., 2017a) using the Illumina HiSeq2000 (Illumina, San Diego, CA, USA) and the data were analyzed using the CLC Genomic Workbench (CLCbio, Aarhus, Denmark) to determine the mutated genes. The *sigA* gene was amplified with Prime STAR Max DNA polymerase using the primers sigA_Fw and sigA_Rev (Table 1), and the mutation site was confirmed by sequencing using an ABI3130 sequencer.

**Table 1:** **Primers used in this study**.

**Purpose**	**Primer name**	**Sequence (5′–3′)**
Amplification of *sigA* gene	sigA_Fw	AAATAAGCATGATCTGAGCC
	sigA_Rev	AATTAAGGGAAGCTACAAGG
Sequencing of *sigA* gene	sigA_seq_Fw	TTTCTTCTGGTGCTGGAT
	sigA_seq_Rev	GTAGGTCGTGGTATGTTATT
Amplification of *fbaA* gene	fbaA_Fw	TGTAGAAACCGCTCATGTAA
	fbaA_Rev	GACATCTTTATCCTCCAATC
Amplification of His-tagged SigA	His_SigA_Fw	CGCGGATCCATGTCTGATAACACAGTTAAA
	His_SigA_Rev	GCGCTCGAGTTAATCCATAAAGTCTTTCAA

### Phage transduction

Phage transduction using phage 80 α was performed as previously described (Novick, 1991). To 100 μL of overnight culture of the recipient bacteria, 200 μL of the donor phage was added, and the solution was mixed with 3 mL of top agar [50% 0.3GL (casamino acid 3g, yeast extract 3g, NaCl 5.9g, 60% sodium lactate syrup 3.3 mL, 25% glycerol 4 mL per liter) and 0.75% agar] and poured into a plate containing bottom agar (50% 0.3GL, 1.5% agar, and 37.5 μg/mL chloramphenicol) and middle agar (50% 0.3GL and 1.5% agar). The plates were grown at 30°C for 2–3 days. The transductants grown on the plates were isolated. The MIC value of GPI0363 against these isolates was determined and the mutation site of the *sigA* gene was identified by sequencing on an ABI3130 sequencer. Colony polymerase chain reaction (PCR) was performed using KOD fx Neo DNA polymerase and primers: sigA_Fw and sigA_Rev (Table 1). The primers used for sequencing were: sigA_Fw, sigA_Rev, sigA_seq_Fw, and sigA_seq_Rev (Table 1).

### Preparation of DNA template and *S. aureus* RNA polymerase holoenzyme

For template preparation, a 406-bp long portion of the *fbaA* gene including its promoter was amplified using Prime STAR max DNA polymerase with the primers fbaA_Rev and fbaA_Fw (Table 1). The obtained PCR product was electrophoresed in 1% agarose and purified by gel extraction (QIAquick Gel Extraction kit 250, Qiagen, Hilden, Germany). *S. aureus* RNA polymerase (RNAP) was partially purified as described previously (Deora and Misra, 1996) with slight modification. Briefly, *S. aureus* strain RN4220 or GPI0363-resistant cells were grown in nutrient broth containing 2% casein enzymatic hydrolysate and 1% yeast extract until OD_600_ was 1.0. The cells were then harvested by centrifugation at 8000 rpm for 10 min at 4°C and washed with buffer A [10 mM Tris-HCl (pH 7.9), 10 mM MgCl_2_, 1.0 M NaCl, 5 mM EDTA (pH 8.0), 0.2 mM dithiothreitol (DTT), and 5% glycerol], followed by grinding buffer (0.05 M Tris, 5% glycerol, 2 mM EDTA, 0.1 mM DTT, 1 mM 2-mercaptoethanol, 0.233 M NaCl, 130 μg/mL lysozyme, 23 μg/mL phenylmethylsulfonyl fluoride). Cells (~2g) were suspended in 10 mL of grinding buffer [0.05M Tris, 5% (v/v) glycerol, 2 mM EDTA, 0.1 mM DTT, 1 mM 2-mercaptoethanol, 0.233 M NaCl, 130 μg/mL lysozyme, and 23 μg/mL phenylmethylsulfonyl fluoride], treated with 1 mg lysostaphin and incubated for 30 min at room temperature. The cells were homogenized in a polytron homogenizer (Kinematica, Littau, Switzerland) and subjected to ultra-centrifugation at 80,000 rpm for 30 min at 4°C. Ammonium sulfate (35 g/100 mL) was added to the resulting supernatant and precipitate was collected by centrifugation at 8000 rpm for 30 min at 4°C, and washed with saturated ammonium sulfate solution. The precipitate was suspended in TGED buffer [10 mM Tris-HCl (pH 7.9), 5% glycerol, 0.1 mM EDTA, and 0.2 mM DTT], aliquoted, and stocked at −80°C until use.

### *In vitro* transcription assay

The *in vitro* transcription assay was performed as previously described (Deora and Misra, 1996) with slight modification. The reaction mixture contained a final concentration of 40 mM Tris acetate (pH 7.9), 100 mM NaCl, 5 mM MgCl_2_, 0.2 mM DTT, 100 μg/mL bovine serum albumin (BSA), 0.25 mM each of ATP, CTP, GTP, 0.015 mM UTP, 10 μci of [α-^32^P] UTP (PerkinElmer, Waltham, MA, USA), 0.5 units of RNase inhibitor, and partially purified *S. aureus* RNAP. GPI0363 was prepared in DMSO for the assay. The total assay concentration of DMSO did not exceed 4%. GPI0363 or DMSO was added to the reaction mixture devoid of nucleoside triphosphates and template DNA, and incubated at room temperature for 10 min. The transcription reaction was started by adding nucleoside triphosphates and template DNA, and further incubated for 10 min at 35°C. The samples were placed on ice, and 100 μL of stop solution (0.4 M ammonium acetate, 20 mM EDTA, 0.3% SDS, 4 μg of tRNA) was added. Transcripts were extracted by phenol chloroform, electrophoresed on 7M urea 6% polyacrylamide gel, and visualized by autoradiography using Typhoon FLA 9000 (GE Healthcare, Tokyo, Japan).

### Purification of his-tagged recombinant SigA

The *sigA* gene was amplified by PCR with Prime STAR max DNA polymerase using primers His_SigA_Fw and His-SigA_Rev from the genomic DNA of *S. aureus* RN4220 (Table 1). The PCR product was digested with BamHI and XhoI, and cloned in the pET28a vector (Novagen). The plasmids were introduced into *Escherichia coli* BL21(DE3)/pLysS, and cells were selected with 50 μg/mL kanamycin. Colonies were inoculated into Luria Bertani medium in the presence of 50 μg/mL kanamycin. After overnight incubation at 37°C, 5 mL of the culture was added to 500 mL of the fresh medium containing 50 μg/mL kanamycin and incubated with shaking at 37°C. After the OD_600_ reached 0.3, 1 mM of isopropyl β-D-1-thiogalactopyranoside was added, and the culture was further incubated for 5 h at 30°C. Cells were collected by centrifugation and frozen by liquid nitrogen. SigA was purified using the Probond^TM^ purification system according to manufacturer's protocol (Life Technologies, Carlsbad, CA, USA).

### Binding of SigA with GPI0363

TALON® magnetic beads pre-charged with cobalt (Clontech Laboratories, Mountain View, CA, USA) were equilibrated with 50 mM Tris buffer, pH 7.5. The equilibrated beads were incubated with His-tagged recombinant SigA in the presence and absence of GPI0363 for 30 min in a rotary shaker. GPI0363 was pretreated with BSA (0.5 mg/mL) prior to incubation. The resulting beads were washed with the same buffer, separated, and eluted with 50% acetonitrile + 0.1% trifluoroacetic acid. The eluted fractions were analyzed by HPLC in TSKgel α-M size exclusion column (7.8 mm ID × 30 cm, 13 μm; TOSOH) with 50% acetonitrile + 0.1% trifluoroacetic acid at a flow rate of 0.5 mL/min.

### Mouse infection model

All mouse protocols were approved by the Animal Use Committee at the Graduate School of Pharmaceutical Science at our previous affiliation at the University of Tokyo. MRSA USA300 was cultivated overnight in TSB at 37°C. The culture was centrifuged at 10,000 rpm for 1 min at 4°C and the pellet was suspended in phosphate-buffered saline (PBS). GPI0363 was dissolved in a mixture of 1:1:6 ethanol/cremophor EL/0.9% NaCl. Mice (ICR, female, 18–20 g, 4 weeks old, CLEA, Tokyo, Japan) were infected with the bacterial suspension of 1.0 × 10^9^ CFU per mouse by intravenous injection, followed by intraperitoneal injection of 400 mg/kg GPI0363 (*n* = 7) 30 min after infection. Control groups were injected with bacteria only (*n* = 8) or vehicle only (*n* = 7). Survival was noted in a condition that all mice of the vehicle group survived. Data were analyzed using Prism 5 for Mac OS X, version 5.0d (GraphPad Software).

## Results

### Screening and identification of *in vivo* effective anti-staphylococcal agent

**Figure 1:**
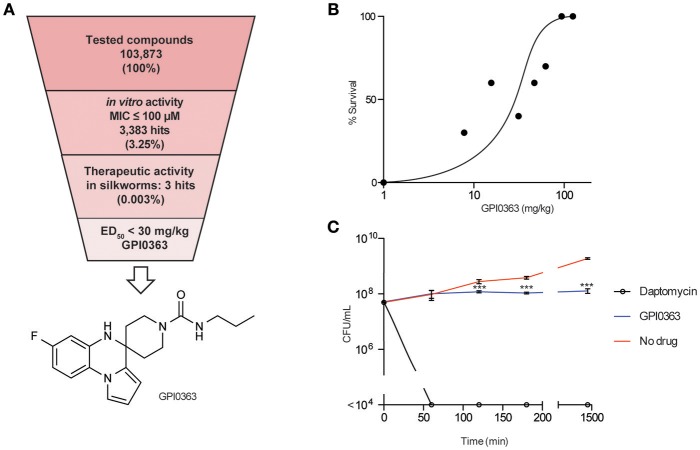
*****In vitro*** and ***in vivo*** activities of GPI0363. (A)** Screening strategy and chemical structure of GPI0363. Compounds that displayed *in vitro* activity against *S. aureus* MRSA4 by broth microdilution assay were screened using the silkworm infection assay. Silkworms (*n* = 3) were injected with *S. aureus* MSSA1 suspension (3 × 10^7^ CFU/larva) and immediately injected with 50 μL of 2 mM compounds. **(B)** *In vivo* activity of GPI0363 in the silkworm infection model. Silkworms were injected with *S. aureus* MSSA1 suspension (3 × 10^7^ CFU/larva) and immediately injected into the hemolymph with GPI0363 at different doses (*n* = 10). **(C)** Bacteriostatic activity of GPI0363. Exponentially growing *S. aureus* MSSA1 was treated with 20 μg/mL GPI0363, 5 μg/mL daptomycin, or vehicle. Culture aliquots were collected at different intervals as shown, diluted, and spread on agar plates, and cell viability was determined by counting the CFU per mL. Data are shown as mean ± *SD* of three independent experiments. Data were analyzed by one-way ANOVA using Dunnett's Multiple Comparison test and significant differences compared with no drug are indicated by asterisks (^***^p ≤ 0.001).

We screened a synthetic chemical library derived from the Drug Discovery Initiative at the University of Tokyo for compounds that inhibit the growth of an MRSA strain. Of 103,873 synthetic compounds, 3383 (3.25%) compounds inhibited the growth of MRSA *in vitro*. Next, we used the silkworm infection model as a secondary screening tool, and identified three (0.003%) compounds with therapeutic activity in silkworms infected with methicillin-susceptible *S. aureus* (MSSA) (Table 2, Figure 1A). Given that current treatment options for MRSA are quite limited and antimicrobial agents that show activity against MRSA exert activity against MSSA, we used MRSA during *in vitro* screening. Further, to examine the therapeutic activity in silkworms, we used MSSA as this system is robust and well-established. We focused on a spiro-heterocyclic compound, GPI0363 (7-fluoro-N-propylspiro[5H-pyrrolo[1,2-a]quinoxaline-4,4′-piperidine]-1′-carboxamide) (Figure 1A), as this was the most potent of the three compounds with an ED_50_ value of 26 mg/kg (Figure 1B, Table 3). GPI0363 was effective against several *Staphylococcus* sp., including MRSA, *S. pseudintermedius*, and *S. haemolyticus*. It was not effective against other tested Gram-positive and Gram-negative bacteria (Table 4). We tested the killing effect of GPI0363 on exponentially growing *S. aureus* and found that treatment of *S. aureus* with GPI0363 did not significantly change the colony-forming capacities after 24 h, suggesting that the action of the compound is bacteriostatic (Figure 1C). We, further found that the rate of appearance of spontaneous resistance to GPI0363 was <10^−7^ (12.5 μg/mL).

**Table 2:** **Screening of chemical library for therapeutically effective anti-staphylococcal agent**.

**Category**	**Parameter**	**Description**
Assay	Type of assay	Inhibition of methicillin-resistant *Staphylococcus aureus* (MRSA) growth
	Assay strategy	Identification of growth inhibitors of *Staphylococcus aureus in vitro*
	Assay protocol	Broth microdilution assay according to CLSI (Clinical and Laboratory Standards Institute, 2012)
	Primary measurement	Minimum Inhibitory Concentration (MIC) against MRSA4
Library	Library size	103,873 screened
	Library composition	Synthetic chemical compounds
	Source	Drug Discovery Initiative, The University of Tokyo
Screen	Format	96-well-plates, round bottom
	Concentration(s) tested	100 μM
	Plate controls	Vancomycin (100 μg/mL) and DMSO
	Reagent/ compound dispensing system	Manual
	Detection instrument and software	Manual
	Hit criteria	Compounds inhibiting growth of *S. aureus* MRSA4 at a concentration of 100 μM
	Hit rate	3,383 (3.25%)
Secondary screen	Additional assay(s)	Screening for therapeutic activity using silkworm infection model infected with *S. aureus*
	Assay strategy	Identification of compounds that cure silkworms from *S. aureus* infection
	Assay protocol	According to Hamamoto et al. (2015). Fifty microliters of the compounds were injected to each silkworm.
	Concentration(s) tested	2 mM
	Hit criteria	Compounds allowing survival of 2 of 3 silkworms infected with *S. aureus* MSSA1
	Hit rate	3 (0.003%)
	Confirmation of hit purity and structure	HPLC, NMR, chemical synthesis, and confirmation of activity of chemically synthesized compound

**Table 3:** *****In vitro*** and ***in vivo*** activities of three candidate hits obtained from secondary screening**.

**Compound**	**MIC (μg/mL)**	**ED_50_ (mg/kg)**
GPI0363	4	26
GPI0235	12.5	45
GPI0262	25	66

**Table 4:** **Antimicrobial spectrum of GPI0363**.

**Bacteria**	**MIC (μg/mL)**
Methicillin-susceptible *Staphylococcus aureus* (MSSA)	
MSSA1 (clinical isolate)^*^	4
NCTC 8325^*^	8
RN4220^*^	4
Newman^*^	4
Smith ATCC13709^*^	4
Methicillin-resistant *S. aureus* (MRSA)	
MRSA4 (clinical isolate)^*^	4
USA300 FPR3757 (clinical isolate)^**^	4
*Staphylococcus haemolyticus* JCM2416^*^	4
*Staphylococcus pseudintermedius* JCM17571^*^	4
*Bacillus subtilis* JCM2499^*^	128
*Bacillus cereus* JCM20037^*^	128
*Listeria monocytogenes* 10403S^*^	128
*Enterococcus faecalis* EF1^***^	>256
Vancomycin resistant *Enterococcus faecalis* EF5^***^	>256
*Streptococcus pneumoniae* (clinical isolate)^*^	>256
*Streptococcus agalactiae* JCM5671^*^	>256
*Streptococcus sanguinis* JCM5708^*^	>256
*Serratia marcescens* (clinical isolate)^*^	>256
*Escherichia coli* W3110^*^	>256
*Pseudomonas aeruginosa* PAO1^*^	>256

*Minimum inhibitory concentration (MIC) was determined against bacteria by broth microdilution assay. The sources of the strains are indicated by asterisks*.

* *Hamamoto et al., 2015*,

** *Suzuki et al., 2011*,

*** *Paudel et al., 2012*.

### GPI0363 inhibits RNA synthesis in *S. aureus*

**Figure 2:**
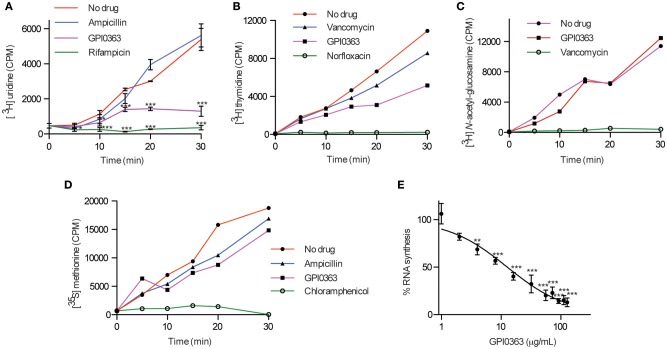
**Effect of GPI0363 on macromolecule biosynthesis**. The effect of GPI0363 on macromolecule biosynthesis was examined by measuring the incorporation of the corresponding radiolabeled precursors. Aliquots were collected at different intervals as indicated, and the radioactivity of acid-insoluble fractions was measured by a liquid scintillation counter and is shown as counts per minute (CPM). **(A)** Effect on RNA synthesis. Data are shown as mean ± *SD* of three independent experiments and analyzed by one-way ANOVA using Dunnett's Multiple Comparison test and significant differences compared with no drug are indicated by asterisks (^*^p ≤ 0.05, ^***^p ≤ 0.001) **(B)** Effect on DNA synthesis. **(C)** Effect on peptidoglycan synthesis. **(D)** Effect on protein synthesis. Data from a single experiment are shown in figures **(B–D)**. **(E)** Dose response of RNA synthesis inhibition: Aliquots were collected after 30 min, and radioactivity of the acid insoluble fractions was measured. Data represents mean ± *SD* of triplicates. Data were analyzed by one-way ANOVA using Dunnett's Multiple Comparison test and significant differences compared with no treatment are indicated by asterisks (^**^p ≤ 0.01, ^***^p ≤ 0.001).

To elucidate the mode of action of GPI0363, we determined its effect on macromolecule biosynthesis by measuring the incorporation of radiolabeled precursors into the acid-insoluble fractions in the presence of the compound. GPI0363 significantly inhibited the incorporation of [^3^H] uridine, a precursor of RNA synthesis, within 30 min in exponentially growing *S. aureus* (Figure 2A). We observed a slight inhibition of the incorporation of [^3^H] thymidine, a precursor of DNA synthesis, and no inhibition of the incorporation of [^3^H] *N*-acetyl-glucosamine and [^35^S] methionine, precursors of peptidoglycan and protein synthesis, respectively (Figures 2B–D). GPI0363 significantly inhibited RNA synthesis in *S. aureus* in a dose-dependent manner, and the half-maximal inhibitory concentration (IC_50_) value was 12 μg/mL (Figure 2E).

### GPI0363-resistant strains harbor a mutation in the *sigA* gene

**Figure 3:**
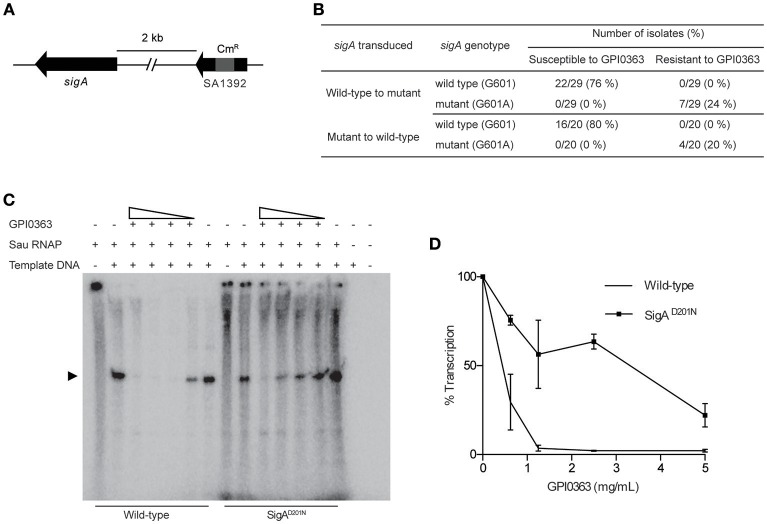
**Involvement of SigA in transcription inhibition by GPI0363. (A)** *sigA* gene and the resistance marker used for the phage transduction experiments. **(B)** Correlation between mutation in the *sigA* gene and resistance to GPI0363 by phage transduction analysis. Wild-type *sigA* gene and mutated *sigA* gene were transduced into the GPI0363-resistant strain and wild-type RN4220 strain, respectively, by phage transduction. The genotype of the *sigA* gene and the susceptibility of the strains toward GPI0363 were determined. **(C)** GPI0363 inhibits promoter-specific transcription *in vitro*. Sau RNAP from wild-type strain and SigA^D201N^ mutant GPI0363-resistant strain were prepared as described in the Section Materials and Methods. Transcripts were extracted and electrophoresed on 7M urea and 6% polyacrylamide gel, and visualized by autoradiography. The promoter-specific transcript band is indicated by the arrowhead in the autoradiogram. **(D)**The intensities of the bands obtained were measured quantitatively using ImageJ software. The band intensity in the lanes containing the fraction and template was taken as 100%. Results are expressed as the average and range of the two independent experiments.

To identify a cellular target of GPI0363, we treated *S. aureus* RN4220, a strain suitable for genetic manipulations (Monk and Foster, 2012), with a mutagen ethyl methanesulfonate and spread it on Luria Bertani agar plates containing 12.5, 25, or 50 μg/mL GPI0363. We observed no colonies on the plate containing 50 μg/mL GPI0363. From the plates containing 12.5 and 25 μg/mL GPI0363, a total of 150 colonies were isolated. Several studies have demonstrated that drug-resistant phenotypes showing temperature sensitivity harbor mutations in genes related to the action of the drug (Canepari et al., 1987; Hamamoto et al., 2015). Among the 150 colonies, we screened for temperature-sensitive phenotypes and checked their susceptibility to GPI0363. We identified two strains with a temperature-sensitive phenotype that displayed resistance to GPI0363 (MIC ≥ 16 μg/mL) (GPI0363^R^74 and GPI0363^R^108) (Table 5). We could not correlate the temperature-sensitive phenotype with resistance to GPI0363; therefore, we sequenced the whole genome of both the strains by a next generation sequencer. Along with other mutations, these strains commonly had a point mutation in the *sigA* gene (G601A) leading to an amino acid substitution, (D201N) (Table 6). Mutation in the same gene in the independent resistant strains led us to speculate the involvement of the *sigA* gene product in the anti-staphylococcal activity of GPI0363. To test whether the *sigA* gene is involved in resistance conferred by GPI0363, we performed genetic recombination with phage transduction experiments (Novick, 1991) using phage 80α. For the first phage transduction, wild-type *sigA* gene was introduced into the GPI0363-resistant mutant and transductants were isolated by chloramphenicol selection. The donor and recipient were phage 80α harboring wild-type *sigA* and a chloramphenicol-resistant (Cm^R^) marker inserted into the SA1392 gene (Figure 3A), and GPI0363-resistant strain GPI0363^R^74, respectively. Next, we introduced the mutated *sigA* gene into the wild-type strain using phage 80α with mutant *sigA* (G601A) and Cm^R^ marker as the donor, and wild-type RN4220 strain as the recipient. The genotype of the transductants was confirmed by sequencing. In both the phage transduction experiments, we found that the transductants harboring the wild-type genotype had restored susceptibility to GPI0363 and strains with a mutant genotype showed resistance to GPI0363 (Figure 3B, Supplementary Tables 1, 2). Thus, we found that resistance to GPI0363 and the mutation in the *sigA* gene were co-transducible, suggesting that the single mutation in the *sigA* gene led to resistance to the compound in these strains. Further, we used BLAST to analyze the sequence of SigA of different bacteria used in our study. We found that the SigA in bacteria that were resistant to GPI0363 had <80% identity to that of staphylococci (Table 7). Hence, the specificity of GPI0363 toward staphylococci could be explained by the differences in SigA among different bacteria.

**Table 5:** **MIC values of GPI0363 against wild-type and EMS treated selected strains**.

**Bacterial strain**	**MIC (μg/mL)**
*S. aureus* RN4220	4
GPI0363^R^74	16
GPI0363^R^108	32

*MIC was determined against listed strains by broth microdilution assay*.

**Table 6:** **GPI0363-resistant strains and the genes mutated**.

**GPI0363^R^74**	**GPI0363^R^108**
*gatA*	SA1708
*uppS*	SA0940
*capI*	*infB*
SA1675	SA0544
*sigA* (G601A)	*sigA* (G601A)
*sirC*	*uvrA*
*tgt*	
*dnaK*	
SA1444	
*adhE*	
*iunH*	
SA0551	
*thiE*	
*yycH*	
*metE*	

*Genomic DNAs were extracted from the resistant strains and mutation in genes were determined by the next generation sequencer. Underlined are the essential genes*.

**Table 7:** **Comparison of staphylococcal SigA with different bacteria**.

**Organism (Accession no.)**	**Similarity (% identities)**
*Staphylococcus aureus* (WP_001283055)	100
*Listeria monocytogenes* 10403S (AEO06439)	78
*Bacillus subtilis* JCM2499 (EFG90416)	79
*Streptococcus pneumoniae* (WP_000818341)	69
*Streptococcus sanguinis* JCM5708 (EGF07840)	67
*Streptococcus agalactiae* JCM5671 (EFV97681)	69
*Enterococcus faecalis* (WP_048946466)	77
*Escherichia coli* W3110 (WP_000437376)	67
*Pseudomonas aeruginosa* PAO1 (AAG03965)	69
*Serratia marcescens* (WP_060388246)	67

### GPI0363 inhibits promoter-specific transcription *in vitro*

The *sigA* gene is an essential gene encoding the primary sigma factor, SigA, of *S. aureus*. SigA binds to the RNAP core enzyme to form the holoenzyme and facilitates recognition of the promoter region, separation of DNA strands, and the initiation of transcription (Burgess et al., 1969; Gross et al., 1988; Helmann and dehaseth, 1999). We partially purified the *S. aureus* RNAP holoenzyme (hereafter referred to as Sau RNAP) from the wild-type strain and GPI0363-resistant strain (SigA^D201N^), and determined the effect of GPI0363 on their transcription ability using a DNA template harboring the *fbaA* gene promoter. GPI0363 inhibited the promoter-specific transcription by wild-type Sau RNAP while the SigA^D201N^ mutant Sau RNAP was comparatively resistant to the compound (Figures 3C,D) with an IC_50_ value of 140 and 650 μg/mL for the wild-type and the SigA^D201N^ mutant Sau RNAP, respectively.

**Figure 4:**
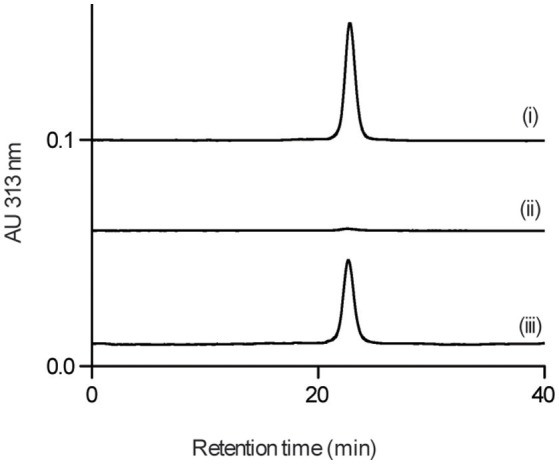
**GPI0363 binds to SigA**. Magnetic beads with (i) and without (ii) His-tagged SigA were incubated with GPI0363 pretreated with BSA; the bound fraction was washed, eluted, and analyzed by HPLC. The retention time of the peak appeared was confirmed to be GPI0363 by running reference GPI0363 (iii). Representative result of two independent experiments is shown.

We next examined whether GPI0363 directly binds to SigA. To establish the binding assay, we purified histidine-tagged recombinant SigA from the wild-type strain. Next, we used magnetic beads with an affinity for histidine and incubated them with His-tagged SigA, followed by incubation with GPI0363 pretreated with BSA. We separated the bound fraction, and then washed, eluted, and analyzed the eluted fractions using HPLC. The peak of GPI0363 appeared when mixed with SigA whereas the peak was not observed when SigA was omitted (Figure 4), suggesting the direct binding of GPI0363 to SigA.

### Activity of GPI0363 in a mouse infection model

**Figure 5:**
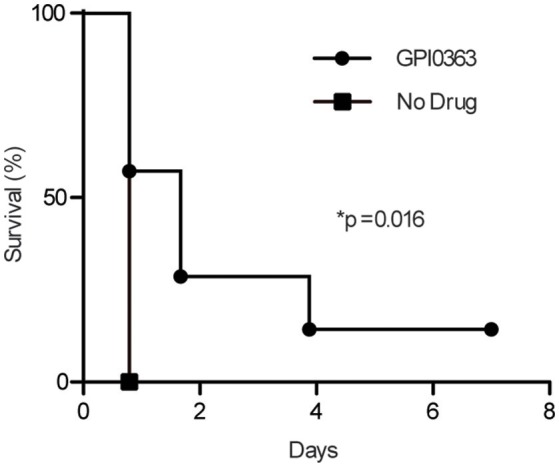
**Effect of GPI0363 on a mouse model of infection**. Mice were infected with *S. aureus* USA300 by intravenous injection, followed 30 min later by intraperitoneal injection of 400 mg/kg GPI0363. Data were analyzed using the Gehan-Breslow-Wilcoxon test (^*^p = 0.016) and log-rank test (^*^p = 0.016).

To determine the effect of GPI0363 in infected mice, mice were injected intravenously with MRSA USA300 followed 30 min later by intraperitoneal administration of 400 mg/kg GPI0363 (*n* = 7). Administration of GPI0363 prolonged the survival of the mice compared to the control (Figure 5). The median survival time of the infection control group was 0.8 days while median survival of the GPI0363-treated group was 1.7 days. Thus, an antimicrobial compound identified using the silkworm model also exhibited activity in a mouse infection model.

## Discussion

In the current condition of the increased incidence of multi-drug resistant organisms due to the overuse of broad spectrum antimicrobials, the use of a narrow-spectrum antimicrobial agent is desired to treat specific infections as a tailor-made therapy. In addition to the increased effectiveness of treatment and the reduced incidence of multidrug resistance, these compounds will have fewer side effects on normal gut flora and the host immune system. Here we screened a chemical library of compounds using silkworms infected with *S. aureus* and identified three potential antimicrobial agents with therapeutic activity. One of these, GPI0363, was active against both MSSA and MRSA, and may be useful to treat severe staphylococcal infections.

Bacterial RNA polymerase comprises of five subunits, α*αββ*'ω, forming the core enzyme. A sigma factor binds to the core enzyme to form the holoenzyme and helps recognize the promoter region to initiate specific transcription (Burgess et al., 1969). The primary sigma factor, SigA, is unique to bacteria, essential for cell viability (Chaudhuri et al., 2009), and responsible for transcription of housekeeping genes during the exponential phase (Deora and Misra, 1996). *S. aureus* SigA differs from the eukaryotic transcription initiation factor TFIID (Horikoshi et al., 1989) and, because the transcription initiation mechanisms in prokaryotes and eukaryotes are entirely different, RNA polymerase is now a validated target for antibacterial agents (Ho et al., 2009). Only two classes of RNA polymerase inhibitors, rifampicin and lipiarmycin, are currently in clinical use (Sonenshein et al., 1974; Tupin et al., 2010). We found that GPI0363 inhibited transcription in *S. aureus* and that the staphylococcal SigA is involved in the mechanism of action of GPI0363. Based on this finding, we speculate that GPI0363 inhibits or alters the formation of the RNA polymerase holoenzyme or the binding to promoter region, or the steps following binding, but the exact mechanism of how GPI0363 inhibits transcription requires further investigation.

Our results further highlight the usefulness of the silkworm infection model as a screening system to eliminate compounds that are effective only *in vitro*. The use of invertebrate animal models for drug screening has many advantages over the use of mammalian models. Several invertebrate animal models and the benefits of silkworms for screening antimicrobial agents have been reviewed elsewhere (Panthee et al., 2017b). The silkworm infection model has been used to identify several novel therapeutically effective antibiotics, such as lysocin E (Hamamoto et al., 2015), nosokomycin (Uchida et al., 2010), and ASP2397 (Nakamura et al., 2017), from the culture broth of microorganisms. One limitation of the silkworm infection model is that the compounds are injected immediately after injection of the bacteria, which does not mimic real-life situations. In addition, antimicrobial compounds that are effective only in mammals might not be identified using the silkworm infection model. Here, GPI0363 protected both silkworm and mice from *S. aureus*-induced infection. These promising results highlight the potential of this drug as a novel therapy for infections caused by *S. aureus*. However, toxicity studies are necessary to support its application in to the clinic.

## Conclusion

The discovery of therapeutically effective antimicrobial agents remains a challenge for drug-development. By using the silkworm infection model for screening, we were able to eliminate compounds that displayed only *in vitro* activity and identified a new anti-staphylococcal agent, GPI0363. GPI0363 inhibited the growth of *S. aureus* by inhibiting RNA synthesis. Our findings suggest the involvement of the primary sigma factor, SigA, in the anti-staphylococcal activity of GPI0363.

## Author contributions

AP, HH, and KS designed research; AP, SP, HH, and KS performed experiments; YS performed genetic analysis; MK, SM, and KK performed chemical synthesis and analysis. AP and HH wrote the manuscript; KS integrated the research and supervised the project. KS and MK critically revised the manuscript for important intellectual content. All the authors commented on and approved the manuscript. KS decided the final approval of the manuscript.

## Funding

This work was supported by JSPS postdoctoral fellowship and TBRF postdoctoral fellowship to AP, MEXT KAKENHI (JP221S0002) and a Grant-in-Aid for challenging Exploratory Research (JP26670043) to HH; and Grant-in-Aid for scientific research (S) (JP15H05783) to KS.

### Conflict of interest statement

KS is a consultant for Genome Pharmaceutical Institute Co., Ltd. The other authors declare that the research was conducted in the absence of any commercial or financial relationships that could be construed as a potential conflict of interest.
