# Supplementary material for: A Novel Spiro-Heterocyclic Compound Identified by the Silkworm Infection Model Inhibits Transcription in Staphylococcus aureus
Source: Front Microbiol. 2017 Apr 25;8:712. doi: 10.3389/fmicb.2017.00712 (PMC5403886; doi:10.3389/fmicb.2017.00712)
Supplement: Supplementary file 1 [file Table1.DOCX]

**Supplementary Information**

**A Novel Spiro-heterocyclic Compound Identified by the Silkworm Infection Model Inhibits Transcription in *Staphylococcus aureus***

Atmika Paudel, Hiroshi Hamamoto, Suresh Panthee, Keiichi Kaneko, Shigeki Matsunaga, Motomu Kanai, Yutaka Suzuki, Kazuhisa Sekimizu

**Supplementary Table S1: Strains obtained after first phage transduction (introduction of wild-type *sigA* to GPI0363-resistant strain)**

| **Strain** | **MIC of GPI0363 (µg/mL)** | **Genotype of *sigA*** |
| --- | --- | --- |
| 1-1 | 4 | G601 |
| 1-2 | 4 | G601 |
| 1-3 | 4 | G601 |
| 1-4 | 4 | G601 |
| 1-5 | 4 | G601 |
| 1-6 | 4 | G601 |
| 1-7 | 4 | G601 |
| 1-8 | 4 | G601 |
| 1-9 | 4 | G601 |
| 1-10 | 4 | G601 |
| 1-11 | 4 | G601 |
| 1-12 | 4 | G601 |
| 1-13 | 4 | G601 |
| 1-14 | 16 | G601A |
| 1-15 | 16 | G601A |
| 1-16 | 16 | G601A |
| 1-17 | 16 | G601A |
| 1-18 | 4 | G601 |
| 1-19 | 4 | G601 |
| 1-20 | 4 | G601 |
| 1-21 | 4 | G601 |
| 1-22 | 4 | G601 |
| 1-23 | 4 | G601 |
| 1-24 | 16 | G601A |
| 1-25 | 4 | G601 |
| 1-26 | 4 | G601 |
| 1-27 | 16 | G601A |
| 1-28 | 16 | G601A |
| 1-29 | 4 | G601 |

**Supplementary Table S2: Strains obtained after second phage transduction (introduction of G601A-*sigA* to wild-type strain)**

| **Strain** | **MIC of GPI0363 (µg/mL)** | **Genotype of *sigA*** |
| --- | --- | --- |
| 2-1 | 16 | G601A |
| 2-2 | 16 | G601A |
| 2-3 | 16 | G601A |
| 2-4 | 4 | G601 |
| 2-5 | 4 | G601 |
| 2-6 | 4 | G601 |
| 2-7 | 4 | G601 |
| 2-8 | 4 | G601 |
| 2-9 | 4 | G601 |
| 2-10 | 4 | G601 |
| 2-11 | 4 | G601 |
| 2-12 | 4 | G601 |
| 2-13 | 4 | G601 |
| 2-14 | 4 | G601 |
| 2-15 | 4 | G601 |
| 2-16 | 4 | G601 |
| 2-17 | 4 | G601 |
| 2-18 | 4 | G601 |
| 2-19 | 4 | G601 |
| 2-20 | 16 | G601A |
